# Canine Lipomas Treated with Steroid Injections: Clinical Findings

**DOI:** 10.1371/journal.pone.0050234

**Published:** 2012-11-30

**Authors:** Barbara Lamagna, Adelaide Greco, Anna Guardascione, Luigi Navas, Manuela Ragozzino, Orlando Paciello, Arturo Brunetti, Leonardo Meomartino

**Affiliations:** 1 Department of Veterinary Clinical Sciences, Unit of Surgery, University of Naples Federico II, Naples, Italy; 2 Department of Biomorphological and Functional Science, University of Naples Federico II, Naples, Italy; 3 Ceinge, Biotecnologie Avanzate, scarl, Naples, Italy; 4 Institute of Biostructure and Bioimaging, CNR, Naples, Italy; 5 Department of Pathology and Animal Health, Unit of Pathology, University of Naples Federico II, Naples, Italy; 6 Interdepartmental Veterinary Radiology Centre, University of Naples Federico II, Naples, Italy; Wake Forest University, School of Medicine, United States of America

## Abstract

Lipomas are common benign tumours of fat cells. In most cases, surgical excision is curative and simple to perform; however, such a procedure requires general anaesthesia and may be associated with delayed wound healing, seroma formation and nerve injury in deep and intramuscular tumours. The objective of this study was to evaluate treatment of subcutaneous, subfascial or intermuscular lipomas using intralesional steroid injections in dogs. Fifteen dogs presenting with lipomas were selected for treatment with ultrasound-guided intralesional injection of triamcinolone acetonide at a dose of 40 mg/mL. Nine subcutaneous and subfascial tumours showed a complete regression. The other lipomas decreased in diameter, achieving, in some cases, remission of discomfort and regression of lameness. Steroid injection was a relatively safe and effective treatment for lipomas in dogs; only six dogs experienced polyuria/polydipsia for about 2 weeks post-treatment.

## Introduction

Lipomas occur approximately in about 16% of dogs [1]. They are benign tumors of fat cells, most common in adult female or elderly obese dogs [2].

Lipomas usually occur as solitary masses, but multiple lipomas can also occur in dogs. These tumours are frequently localised in the subcutaneous tissues but may extend intramuscularly or along deep fascial planes; a non-infiltrative lipoma has been described that can occur intermuscularly in the caudal thigh region [3] or in the thoracic limb, almost in the axilla [4]. Deep lipomas often have a paratesticular location [5]. They have occasionally been described within the thorax and the abdomen, causing clinical signs associated with organ compression [6]–[8]. The occurrence of a thymolipoma has been reported in one dog [9] and of thymofibrolipomas in two dogs [10]. Infiltrative lipomas have also been recognised in dogs, appearing histologically as benign adipocytes invading the muscle, the fascia, and occasionally the bone [11]–[14]. Diagnosis of lipoma is provided only by cytology or biopsy with histological evaluation [1].

Many lipomas are asymptomatic, can be diagnosed with cytological examination and do not require any treatment; however, some tumours may elicit discomfort in dogs due to nerve compression, or, especially if they become large enough, cause functional difficulties [1]. One report described a case of a dog exhibiting lameness secondary to the carpal canal compression [15]. Usually, owners concerned about the growth and appearance of tumours and discomfort to their animals request to have lipomas removed.

In most cases, surgical excision is curative and simple to perform; however, it requires general anaesthesia and may be associated with the risk of delayed wound healing, seroma formation and nerve injury in deep and intermuscular lesions. It has been reported that intralesional injections of 10% calcium chloride solution cause lipoma regression, but this treatment is not recommended because irritation and skin necrosis can occur [16]. Minimally invasive liposuction of a giant lipoma in one dog [17], and irradiation of infiltrative lipomas in 13 dogs have been reported [18]; however, these procedures require general anaesthesia and special devices. In a recent retrospective study of 20 dogs, dry liposuction was effective in preventive the growth of existing lipomas less than 15 cm in diameter, while giant lipomas associated with fibrous trabeculae could not be easily removed and attempts at removal resulted in a high risk of bruising, hematoma and seroma; furthermore, regrowth of tumours was observed in 28% of lipomas treated with liposuction at follow-up between 9 and 36 months [19].

Recent clinical experiences indicate that the use of subcutaneous deoxycholate injections may be a relatively safe and effective treatment for small lipomas, although controlled clinical trials are necessary to substantiate these observations [20]. Recently, the use of a subdermal 1064-nm Nd: YAG laser resulted in complete or almost complete removal of lipoma in 100% of patients [21]. In human patients, there is only about one report that describes intralesional injection of steroids to treat lipomas non invasively [22]. The present study was designed to evaluate the results of treatment of subcutaneous, subfascial, intermuscular or infiltrative lipoma in dogs using steroidal intralesional injections.

## Methods

Our study was reviewed by the committee for experimental animal health at the University of Naples Federico II. The committee stated that the protocol was configured as a Veterinary Medical Diagnostic clinical trial as described in paragraph 3 of article 7 of DL 116/92, which refers to legal obligations of the investigators. Therefore, it was not submitted to the Ministry of Health that normally accompanies each experimental protocol but it was presented as summary activity to the Public Local Health Utility Napoli 1 Center. We confirmed that all animal procedures in this study were conducted by a veterinarian and conformed to all regulations protecting animals used for research purposes, including national guidelines (DL 116/92) as well as the protocols recommended by Workman, et al. (1998).

We enrolled dogs not treated previously for lipoma whose owners sought an alternative treatment to surgery. All dogs underwent laboratory tests to determine their general medical condition. No dogs selected presented with major clinical signs of illness. Written informed consent was obtained from each owner. No anaesthesia or sedation was necessary during the procedures, and no signs of discomfort were observed in dogs during the treatment.

Fifteen dogs referred with subcutaneous, subfascial or intermuscular lipomas and one infiltrative lipoma were selected for this study.

The diagnosis of lipoma was made in all cases on the basis of a physical examination (superficial, well-circumscribed soft mass), ultrasonographic features of the mass (oval or irregularly pedunculated shape, generally with a defined capsule, and with a homogeneous thin striped echo-pattern) and confirmed with Fine Needle Aspiration cytology (FNAC).

For the cytological examination, the samples were spread on glass slides, air-dried and stained with a May-Gruwald Quick stain (Bio-Optica, Milano). The slides were observed with an optical microscope (Nikon, E600) at different magnifications. Ultrasound examination was performed with an 11-MHz linear transducer with a footprint of 4 cm and a scan depth of 6 cm (Logiq MD400, General Electric).

Lipomas were injected with triamcinolone acetonide 40 mg/ml (kenacort®; Bristol-Myers Squibb). A 1-mL or 2-mL syringe with a 22-gauge needle was used to inject the solution transcutaneously at the centre of the lipoma guided by ultrasound. The volume of the steroid injection depended on the size of the lesion: 0.5 mL for small lipomas (<3 cm in diameter); 1 mL for large lipomas (>3 cm in diameter). When the lipoma measured more than 3 cm, two injections of 0.5 mL were made in two sites along the major axis of the lesion. If there were no signs of lipoma regression, the procedure was repeated one month later.

Measurements of tumours were performed by ultrasound before treatment, and 1 and 6 months later. If complications occurred after treatment, they were recorded by the owners and reported at the subsequent visit.

## Results

A total of 15 lipomas (9 subcutaneous, 3 subfascial, 2 intermuscular, 1 infiltrative) in 15 dogs (9 mixed, 6 purebred dogs, mean age 8 year, 4 male, 11 female) were injected with 0.5 mL (10 cases) or 1 mL (5 cases) of triamcinolone acetonide (Tab 1).

For all cases, diagnoses of lipomas originally identified by clinical evaluation and ultrasound were confirmed by cytological examination; lipomas showed histological evidence of normal adipocities on a proteinaceous, bluish background, and, in some cases, aggregated around a blood vessel.

**Figure 1:**
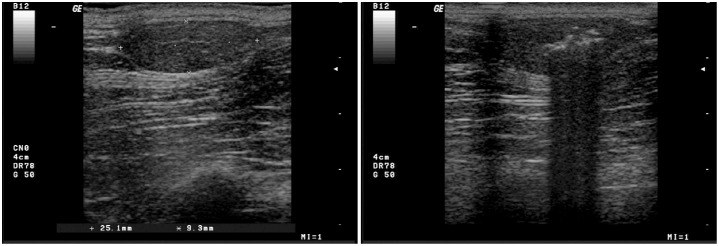
Ultrasound images of an untreated and a treated lipoma. **A** - An oval sub-fascial lipoma in the right axillary region. A well defined hyperechoic capsule is visible; a hypoechoic homogeneous echostructure interrupted by thin stripes is observed. **B** – The lipoma after infiltration of the steroid solution.

After one injection, nine lipomas (six subcutaneous and three subfascial) regressed completely by 6 months follow up. Before the steroid injection, the above-mentioned lipomas showed the following ultrasound features: a hyperechoic capsule, a poorly vascularised hypoechoic or isoechoic echotexture (1 case) with thin hyperechoic stripes homogeneously distributed throughout (fig. 1).

**Figure 2:**
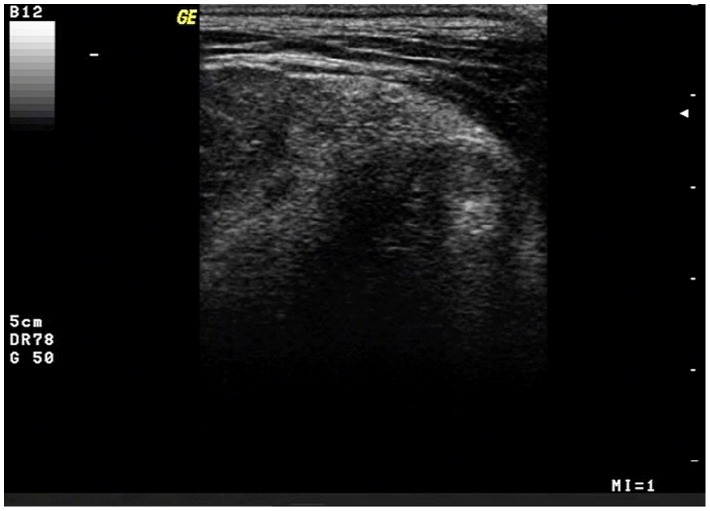
Ultrasound image of an intermuscolar lipoma. An intermuscular lipoma of the caudal region, ultrasonographically appearing hyperechoic and heterogeneous; the capsule is clearly appreciable.

One intermuscular lipoma (dog no. 6) regressed completely after one steroid injection, but, after nine months, recurred, although softer and without any lameness (Table 1). Sonographically, this lipoma appeared hyperechoic, moderately dishomogeneous with a well defined capsule (fig. 2).

**Table 1:** Steroid injections in 15 dogs.

Dog No Breed	Weight Kg, sex	Age years	Site (clinical signs)	Pre-treatment major diameter (mm)	First injection Triamcinolone (mg)	Postreatment major diameter (mm) after 1 month	Second injection Triamcinolone (mg)	Side Effects
**1** Mixed	15 obese, F	7	Right Flank Subcutaneus	25.5	20 mg	Regressed after 1 week	No	Polyuria/polydipsia
**2** Schnautzer	35, F	14	Ventral Neck Subcutaneous	49.9	40 mg	4.9×28.5	40 mg	None
**3** Pit bull	37 obese, M	8	Right Axillary Subfascial	25.1	20 mg	Regressed after 1 week	No	None
**4** Yorkshire	5, F	10	Left Perineal Infiltrative (discomfort)	70.3	40 mg (in two sites)	62 (more soft after 1 week resolution of discomfort)	20 mg (in two sites)	Polyuria/polydipsia
**5** German Shepherd	24, F	3	Right Lumbar Subfascial	43	40 mg	Regressed after 1 week	No	None
**6** Mixed	17 obese, F	14	Left rump intermuscular Gluteal MM (Lamenes)	45.5	40 mg (in two sites)	Regressed for 9 months then recurred more soft (resolution of lameness)	No	
**7** Dalmatian	20, Mn	13	Xiphoid Subcutaneous	29.7	20 mg	Regressed after 1 week	No	None
**8** Mixed	18 obese, Fn	6	Xiphoid Subcutaneous	45	20 mg	27 (Unchanged after 1 year)	No (Owners refused)	Polyuria/polydipsia
**9 Mixed**	15, Fn	10	Inguinal Subcutaneous	38.5	20 mg	19	No (Owners refused)	Polyuria/polydipsia
**10** Rottweiler	45, M	6	Right Flank Subcutaneous	25	20 mg	Regressed after 1 week		None
**11** Mixed	25, Fn	7	Left Lumbar Subfascial	30	20 mg	Regressed after 1 week		None
**12** Mixed	15 obese, F	7	Rigt rump Intermuscolar Gluteal MM (Lamenes)	50.5	40 mg (in two sites)	30.3 (More soft. After 1 week resolution of lameness)	20 mg (in two sites)	Polyuria/polydipsia
**13** Mixed	15 obese, Fn	8	Right Flank Subcutaneous	24.8	20 mg	Regressed after 1 week	No	Polyuria/polydipsia
**14** Mixed	28, M	8	Ventral Neck Subcutaneous	28.2	20 mg	Regressed after 1 week	No	None
**15** Mixed	23, M	10	Left Flank Subcutaneous	27.4	20 mg	Regressed after 1 week	No	None

Note: (F = female, Fn = neutered female, M = male, Mn = neutered male).

Another intermuscular lipoma (dog no. 12) after the first injection partially reduced its size and consistence, with regression of lameness, while a second steroid injection produced further reduction (to less than 50% of the original size).This lipoma was sonographically characterized by a poor vascularized hypoechoic (in comparison with muscles) echotexture, and it appeared to be lobulated.

Larger tumors (subcutaneous tumors located on the neck, on the xiphoid region and on the inguinal region) were partially reduced in size and consistency after the first injection. In the lipoma located on the neck the second steroid injection produced further reduction (to less than 50% of the original size), but after one year, the lipoma returned to its original size. Upon ultrasound examination, the mass appeared isoechoic to the surroundings fat tissues, capsulated, and with a homogeneous thin hyperechoic striped echotexture interrupted by a hypoechoic core showing a mild peripheral vascularity. After 2 years, the lipoma exhibited no alterations in its consistency or size.

**Figure 3:**
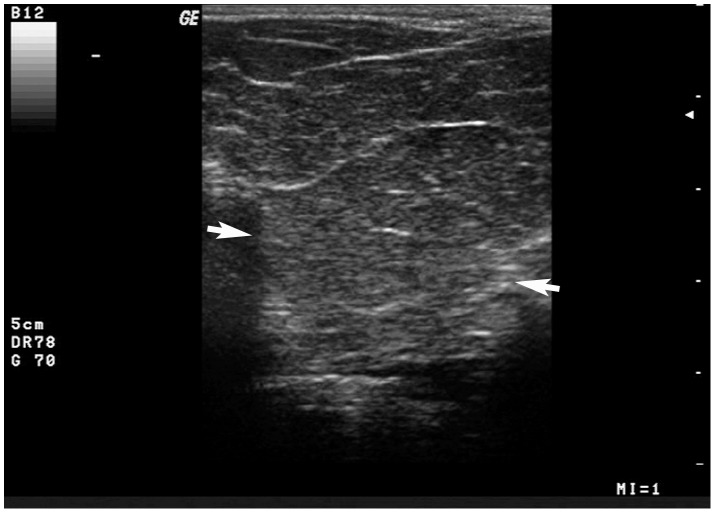
Ultrasound image of perineal lipoma. An infiltrative perineal lipoma, ultrasonographically appearing hypoechoic and multilobulated because of thin hyperechoic lines; a thin limiting capsule is visible (arrows).

An infiltrative perineal lipoma was reduced in size by 70% and in firmness after a second steroid injection, but exhibited a mild increase in size after 10 months. According to the owners, the dog was finally treated with surgical excision of the mass. At surgery, an infiltration of the lipoma into the surrounding tissues was evident. After 2 years post-surgery, the lipoma recurred, but at this time the owners refused further treatment. Upon ultrasound examination, the infiltrative lipoma appeared to be hypoechoic and multilobulated due to the presence of hyperechoic intralesional lines; the tumour displayed a thin capsule poorly defined due to deep pedunculations (fig. 3).

After the first injection of triamcinolone, only six dogs showed secondary effects of polyuria/polydipsia for about 15 days. We observed temporary polyuria/polydipsia in one dog with a weight of less than 8 Kg (small sized dogs) and in five dogs with weights between 8 Kg and 20 Kg (medium sized dogs). No side effects were observed in dogs with weights greater than 20 Kg (large sized dogs) using the described dosage. Treated dogs showed no signs of discomfort.

## Discussion

Steroid injections have the potential to be a safe and effective treatment for small lipomas in dogs. We demonstrated that small lipomas (<3 cm in diameter) and also one of the larger lipomas (>3 cm in diameter) regressed completely after the treatment. The remaining large lipomas (3 subcutaneous, 1 infiltrative, and 2 intermuscular) became less firm and decreased in size. Larger tumours in this study responded less successfully to treatment than smaller tumours, most likely due to the inability of the drug to be distributed throughout the tumour. We recommend that patients with this type of larger tumour undergo surgery. When comparing various available treatments, surgery is generally considered the gold standard, but it has disadvantages, such as the requirement of general anaesthesia.

In our experience, local steroid injection guided by ultrasound achieves reduction of discomfort in dogs with large lipomas and never requires sedation. This treatment could be considered an alternative to surgical excision in older animals that are not candidates for general anaesthesia or deep sedation, and for animals presenting at shows. Previous descriptions of the ultrasonographic appearance of superficial lipomas in dogs [5], [23], [24] are in accordance with our findings. Usually, the ultrasonographic appearance of a lipoma is quite characteristic and easily recognisable as a homogeneous, hypo-isoechoic, capsulated mass, possibly lobulated by thin hyperechoic septa. However, in our sample, one of the intermuscular lipomas displayed a hyperechoic heterogeneous echotexture not common in lipomas. In a report of five cases of infiltrative lipomas in dogs, the masses displayed different echogenicities and echotextures [11], and authors stated that ultrasound was not able to diagnose and visualise the full extent of infiltrative lipomas. Therefore, we conclude that ultrasound examination may have a lower specificity in diagnosing infiltrative and intermuscular lipomas.

It is well known that other drugs such as insulin, antibiotics and methothrexate could also cause localised lipodystrophy in the injection site [25]. The exact mechanism behind the regression of tumours after steroid injections is still unknown. Certain authors reported on the cases of two patients with localised involutional lipoatrophy [26]. These patients received intramuscular steroid injections and immunohistochemical studies with anti-macrophage antibodies (anti-CD68 antigen) showed positive cells scattered around blood vessels and shrunken lipocytes in subcutaneous tissues. Yamamoto T et al (2002) reported on the cases of six patients with localised lipoatrophy characterised by a depressed plaque at the injection sites of corticosteroids [27]. In another report, a case of involutional lipoatrophy was observed post-steroid injection in a 53-year-old woman. Ultrastructural evidence of macrophages in close proximity to altered adipocytes was observed; in addition, macrophages displayed an activated phenotype and were observed engulfing segments of altered adipose and stromal tissue [28].

On the basis of the aforementioned studies, we postulated that steroid injection in the canine lipoma could stimulate an inflammatory response with secondary macrophage activation and production of cytokines. However, to assess the exact mechanism of canine lipid atrophy, it could be useful to perform serial biopsies during the treatment.

Our investigation was conducted choosing an average dosage of triamcinolone acetonide related to the dosage recommended in human patients and derived based on the size of the lipoma [22]. It would be useful to identify the minimum effective dose of steroids on the basis of body weight or body surface area units to prevent side effects in small-breed dogs. Perhaps, in order to reduce complications in dogs with weights less than 20 Kg, several monthly injections of smaller volumes of steroid could be safer than a single injection of steroid as we tested in our protocol.

## Conclusions

Although the exact mechanism of action is still unclear, it appears that steroidal intra-lesional injections may constitute a safe and effective treatment for small collections of fat in dogs.

To prevent any complications, it would be useful to calculate the volume of drug based on the size of dogs and the size of the lipoma.
